# Voltage Dependent Anion Channel Is Redistributed during Japanese Encephalitis Virus Infection of Insect Cells

**DOI:** 10.1155/2014/976015

**Published:** 2014-07-10

**Authors:** Chanida Fongsaran, Narumon Phaonakrop, Sittiruk Roytrakul, Chutima Thepparit, Atichat Kuadkitkan, Duncan R. Smith

**Affiliations:** ^1^Institute of Molecular Biosciences, Mahidol University, Salaya Campus, 25/25 Phuttamonthol Sai 4 Road, Salaya, Nakhon Pathom 73170, Thailand; ^2^Proteomics Research Laboratory, Genome Institute, National Science and Technology Development Agency, 113 Thailand Science Park, Phahonyothin Road, Khlong Nueng, Khlong Luang, Pathum Thani 12120, Thailand; ^3^Center for Emerging and Neglected Infectious Diseases, Mahidol University, 25/25 Phuttamonthon 4 Road, Salaya, Nakhon Pathom 73170, Thailand

## Abstract

Despite the availability of an effective vaccine, Japanese encephalitis remains a significant cause of morbidity and mortality in many parts of Asia. Japanese encephalitis is caused by the Japanese encephalitis virus (JEV), a mosquito transmitted flavivirus. Many of the details of the virus replication cycle in mosquito cells remain unknown. This study sought to determine whether GRP78, a well-characterized flavivirus E protein interacting protein, interacted with JEV E protein in insect cells, and whether this interaction was mediated at the cell surface. GRP78 was shown to interact with JEV E protein by coimmunoprecipitation, and was additionally shown to interact with voltage dependent anion protein (VDAC) through the same methodology. Antibody inhibition experiments showed that neither GRP78 nor VDAC played a role in JEV internalization to insect cells. Interestingly, VDAC was shown to be significantly relocalized in response to JEV infection, and significant levels of colocalization between VDAC and GRP78 and VDAC and ribosomal L28 protein were seen in JEV infected but not uninfected cells. This is the first report of relocalization of VDAC in response to JEV infection and suggests that this may be a part of the JEV replication strategy in insect cells.

## 1. Introduction

Japanese encephalitis virus (JEV) is a mosquito transmitted virus of the genus* Flavivirus*, family Flaviviridae [1]. The genome of JEV is a 5′ capped, single-stranded, positive-sense RNA molecule of approximately 11 kb [2] with a single open reading frame that encodes three structural proteins and seven nonstructural proteins required for viral replication [3]. JEV is distributed in much of Asia and Northern Australia, with most cases of human infection occurring in China, India, and Southeast Asia [4, 5]. JEV is maintained in a natural enzootic cycle between water birds and mosquitoes, although in rural areas pigs are an important amplifying reservoir [4, 6].* Culex tritaeniorhynchus* mosquitoes are the primary transmission vector in India and Southeast Asian countries [7], and vertical transmission of JEV has been reported for this and other mosquito species [4, 6]. Humans can be infected when bitten by an infected mosquito, but humans are a dead end host for virus transmission due to low levels of viraemia [4]. While the majority of cases of human infection with JEV are believed to be asymptomatic [8], an estimated 30,000 to 50,000 cases of Japanese encephalitis (JE) occur annually, resulting in some 10,000 to 15,000 deaths [4, 9, 10].

JEV enters into mammalian cells by at least two different methods of endocytosis. The virus is apparently internalized through a clathrin independent mechanism in cells of a neuronal origin [11, 12], while cells of a nonneuronal origin apparently internalize the virus through clathrin dependent mechanisms [11, 13, 14]. A number of proteins including hsp70 [15, 16], vimentin [17], the low density lipoprotein receptor [18], and CD14 and CD4 [19] have been implicated in playing a role as an initial JEV binding or receptor protein mediating the attachment and internalization of JEV to a variety of mammalian cells.

Significantly less is known about the events mediating JEV entry and replication in insect cells. Susceptible mosquitoes become infected after a blood meal from a viraemic animal [4], although the basis of mosquito susceptibility remains unknown. It is likely however that as with other flavivirus/susceptible host mosquito systems, vectorial competence is determined by up to three genetically determined barriers, namely, the midgut barrier, a midgut escape barrier, and a salivary escape barrier [20]. Studies have suggested that JEV enters into insect cells by endocytosis [21] and hsc70 has been proposed as a putative receptor molecule [22]. Both protein elements and glycosaminoglycans have been implicated in the entry of JEV into insect cells [23].

GRP78 is a multifunctional chaperone protein that has been shown to interact with dengue virus E protein in a number of studies [24–26]. GRP78 is predominantly localized to the endoplasmic reticulum (ER) where it regulates the activation of the unfolded protein response (UPR) in response to stress conditions [27–29]. However, cell surface GRP78 expression is well documented [24, 30–33], and GRP78 has been implicated as a virus receptor for dengue [24, 34] and coxsackievirus A9 [35]. This study sought to initially determine if there was an interaction between GRP78 or the GRP78 interacting protein VDAC (voltage dependent anion channel) and JEV E protein in insect cells and to determine whether the interaction had any relevance to virus internalization.

## 2. Material and Methods

### 2.1. Cell and Virus

The* Aedes albopictus* cell line C6/36 was cultured at 28°C in minimum essential medium (MEM, Gibco Invitrogen, Carlsbad, CA) supplemented with 10% heat-inactivated fetal bovine serum (FBS, Gibco Invitrogen) and 100 units/mL of penicillin and 100 *μ*g/mL of streptomycin (PAA Laboratories, Linz, Austria). LLC-MK_2_ cells (rhesus monkey kidney cell line) were cultured in Dulbecco's modified eagle's medium (DMEM, Gibco Invitrogen) supplemented with 5% FBS with the same antibiotics at 37°C in humidified incubator with 5% CO_2_. The Japanese encephalitis virus strain Beijing-1 (accession number L48961) was propagated in C6/36 cells as described elsewhere [23] and virus stock was prepared by partial purification by centrifugation to remove cell debris and supplementation with 20% FBS before storage at −80°C. Virus titer was determined by standard plaque assay using LLC-MK_2_ cells as described previously [36].

### 2.2. Virus Infection

C6/36 cells were grown under standard conditions until they reached confluence after which they were incubated with JEV in MEM media at the indicated multiplicity of infection (m.o.i.) for 2 hrs, after which the cells were washed with PBS to remove uninternalized virus before being incubated again under standard conditions for the times indicated.

### 2.3. Coimmunoprecipitation Assay

C6/36 cells were grown to 80% confluence in 100 mm^2^ tissue culture plates and either not infected or infected with JEV at MOI of 10 at 28°C for 2 h. Cells were cultured under standard conditions for 3 days after infection after which the cells were collected by centrifugation, washed with PBS, and lysed using Co-IP lysis buffer (25 mM Tris-HCl pH 7.4, 150 mM NaCl, 1 mM EDTA, 1% NP-40, 5% glycerol, 1 mM PMSF, and 1 mM Na_3_VO_4_) followed by incubation on ice for 5 min before centrifugation at 16000 ×g for 5 min. The protein concentration was determined by the Bradford method [37]. To preclear the lysates, 1 mg equivalent of lysates was diluted 2 : 3 and incubated with Protein G Sepharose 4 Fast Flow (GE Healthcare, Buckinghamshire, UK) at 4°C on a rotator for 2 hrs. Subsequently, 100 *μ*L of precleared lysates were incubated with 1 *μ*g of a rabbit monoclonal anti-VDAC antibody or a 1 : 10 dilution of monoclonal antibody HB112 with gentle rocking overnight at 4°C. To precipitate protein complexes, 20 *μ*L of protein G was added to the solutions, which were incubated with gentle rocking at 4°C for 4 hrs. The mixtures were collected by centrifugation at 6000 ×g for 5 min and the supernatants were discarded. The pellets were washed twice with CoIP-lysis buffer and resuspended in 30 *μ*L of 3xSDS sample loading buffer and the proteins were heated to 100°C for 5 min followed by centrifugation at 14000 ×g for 2 min twice before applying the samples to 12% SDS-polyacrylamide gels for subsequent western blot analysis.

### 2.4. Western Blotting

Coimmunoprecipitated proteins were separated by electrophoresis through 12% SDS-polyacrylamide gels and subsequently transferred to solid support (nitrocellulose membranes) using the Trans-Blot electrophoretic transfer cell (Bio-Rad Laboratories, Richmond, CA). The membranes were blocked with 5% skimmed milk in TBS at room temperature for 2 h. For western blot analysis, the membranes were incubated with a 1 : 1000 dilution of a goat polyclonal anti-GRP78 antibody (SC-1050; Santa Cruz Biotechnology Inc., Santa Cruz, CA) followed by a 1 : 2000 dilution of a HRP-conjugated rabbit anti-goat IgG antibody (31402, Pierce, Thermo Fisher Scientific Inc., Rockford, IL) at room temperature for 2 h. Final signal was detected by using the ECL Plus Western Blotting analysis kit (GE Healthcare).

### 2.5. Antibody Mediated Infection Inhibition Assay

C6/36 cells were grown in six-well plates and the cells were then incubated with 20 *μ*g of a rabbit polyclonal anti-VDAC antibody (sc-98708; Santa Cruz Biotechnology Inc.) or 20 *μ*g of a goat polyclonal anti-GRP78 antibody (sc-1050; Santa Cruz Biotechnology Inc.) or 20 *μ*g of a combination of each antibody or 20 *μ*g of a mouse monoclonal anti-*α*V/*β*5 integrin antibody (sc-13588; Santa Cruz Biotechnology Inc.) or no antibody at 28°C for 1. After incubation, the cells were incubated with JEV at MOI of 10 for 2 h at 28°C. The cells were washed with 1x  PBS and treated with acid glycine, pH 3, for 1 min to remove uninternalized virus [38]. The cells were washed with 1x  PBS and fresh growth medium was added. Cells were collected at 8 hours after infection and analyzed by flow cytometry as described previously [19]. Each sample was analyzed in duplicate and the experiment was done independently in triplicate.

### 2.6. Confocal Microscopy and Image Analysis

For intracellular colocalization analysis cells were grown to 60% confluence on glass coverslips before being infected with JEV at m.o.i. 10 for 2 hrs and further cultured under standard protocol. At 24 hrs postinfection cells were fixed with ice-cold absolute methanol for 20 min, washed with PBS, and permeabilized by incubation with 0.3% Triton X-100 in PBS for 10 min before blocking with 5% BSA in PBS for 1 hr. Cells were subsequently incubated with three different primary antibodies simultaneously overnight at 4°C after which cells were washed two times with PBS and incubated with three appropriate secondary antibodies for 1 hr at room temperature before examination under an Olympus FluoView 1000 microscope equipped with Olympus FluoView software version 1.6.

Primary antibodies used were a 1 : 10 dilution of a pan-specific antiflavivirus E protein monoclonal antibody (HB112), a 1 : 50 dilution of rabbit polyclonal anti-VDAC antibody (sc-98708; Santa Cruz Biotechnology Inc.), a 1 : 50 dilution of goat polyclonal anti-GRP78 antibody (sc-1050; Santa Cruz Biotechnology Inc.), and a 1 : 50 dilution of goat polyclonal antiribosomal protein L-28 antibody (sc-14151; Santa Cruz Biotechnology Inc.). Secondary antibodies used were a 1 : 100 dilution of an Alexa 488-conjugated donkey anti-mouse IgG antibody (A21202; Molecular Probes, Thermo Fisher Scientific Inc.), a 1 : 100 dilution of an Alexa 647-conjugated donkey anti-rabbit IgG antibody (A31573; Molecular Probes), a 1 : 100 dilution of an Alexa 568-conjugated donkey anti-goat IgG antibody (A11057; Molecular Probes), a 1 : 200 dilution of an Alexa 594-conjugated chicken anti-mouse IgG antibody (A21201; Molecular Probes), a 1 : 50 dilution of a FITC-conjugated donkey anti-rabbit IgG antibody (sc-2090; Santa Cruz Biotechnology Inc.), and a 1 : 100 dilution of a Cy5-conjugated rabbit anti-goat IgG antibody (81-1616; Invitrogen, Thermo Fisher Scientific Inc.).

Image analysis was undertaken as described previously [39] using the ImageJ analysis program [40] and the PSC colocalization plugin [41]. At least 20 cells were analyzed for each condition. Results are presented in terms of the Pearson correlation coefficients, with statistical analysis of significance between datasets undertaken by a paired sample test using SPSS (SPSS Inc.).

## 3. Results

**Figure 1:**
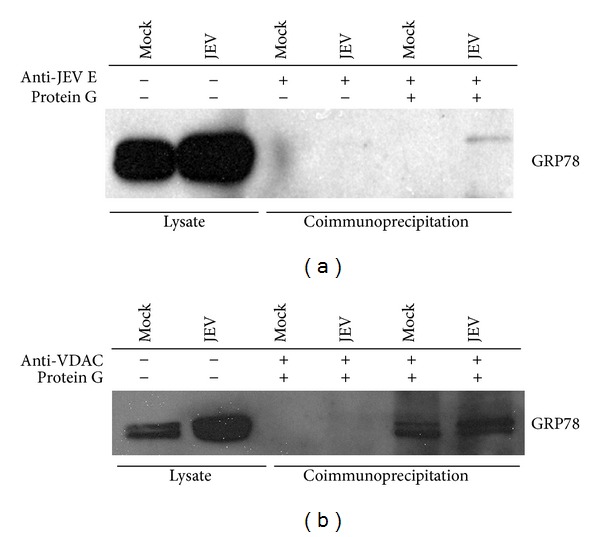
Coimmunoprecipitation analysis of GRP78 and JEV E protein and GRP78 and VDAC. C6/36 cells were mock and or infected with JEV for V days, after which cells were lysed and either E protein (a) or VDAC (b) immunoprecipitated with an appropriate antibody. Coimmunoprecipitation of GRP78 was determined by electrophoresis of the precipitated proteins and transfered to solid matrix support after which membrane was probed with an antibody against GRP78.

To determine whether GRP78 interacts with JEV E protein in insect cells, C6/36 cells were mock infected or infected with JEV and at 3 days after infection JEV E protein was pulled down from the cell lysates using an antiflavivirus E protein monoclonal antibody. The pulled-down proteins were separated by electrophoresis and transferred to solid matrix and the membrane subsequently probed with an anti-GRP78 polyclonal antibody. Results (Figure 1(a)) showed that GRP78 was pulled down in complex with JEV E protein.

Studies in mammalian cells have shown that GRP78 interacts with VDAC at the cell surface [42–44], and so it was determined whether there was an interaction between GRP78 and VDAC in insect cells. C6/36 cells were either mock infected or infected with JEV, and VDAC precipitated with an anti-VDAC antibody and the precipitated proteins were again separated by electrophoresis, transferred to solid support, and probed with an anti-GRP78 antibody. Results (Figure 1(b)) showed that GRP78 was coprecipitated with VDAC. The interaction between GRP78 and VDAC was seen in both infected and uninfected cells, as would be expected, while the interaction between GRP78 and JEV E protein was confined to infected cells. Repeated pull-down experiments failed to demonstrate an interaction between JEV E protein and VDAC (data not shown).

**Figure 2:**
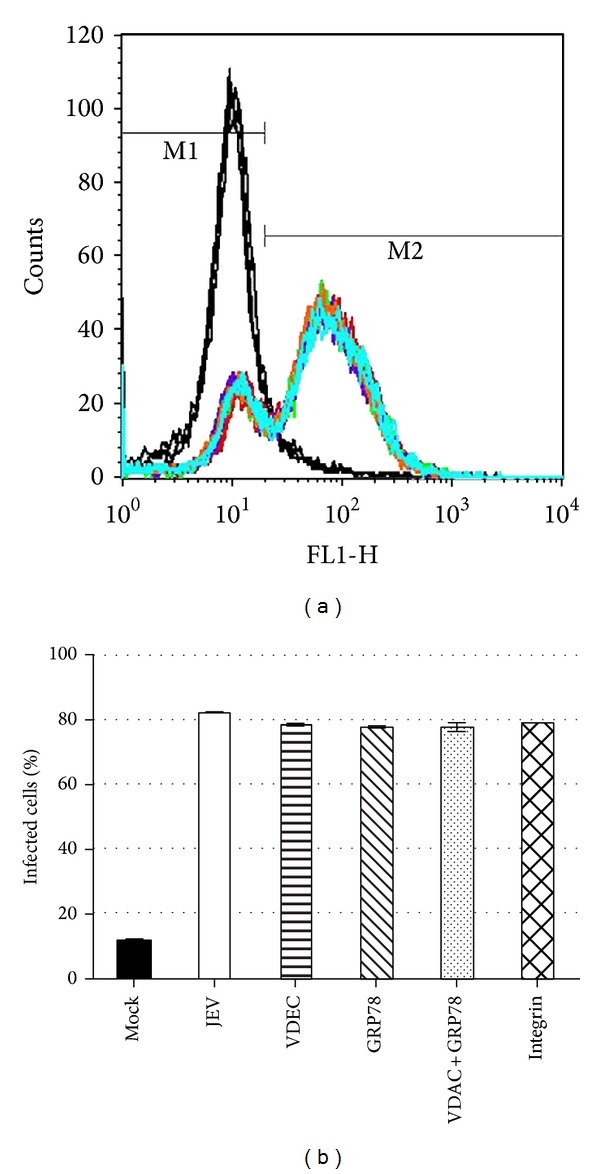
Antibody inhibition analysis of JEV entry to insect cells. C6/36 cells were pretreated with antibodies against GRP78, VDAC, VDAC, and GRP78 and integrin *α*V/*β*5 or no antibody before infection with JEV at MOI of 10. After 8 hours the percent infection was determined by flow cytometry. Representative raw data profiles (a) and tabular analysis (b) from three independent experiments in duplicate are shown.

We next determined whether either of these two proteins (VDAC or GRP78) was acting as JEV receptor proteins through antibody inhibition experiments. C6/36 cells were either mock infected or infected with JEV or infected with JEV after incubation with antibodies directed against VDAC, GRP78, GRP78, and VDAC combined or *α*V/*β*5 integrin as an irrelevant control antibody. Results (Figure 2) showed no inhibition of virus entry under any of the conditions tested.

**Figure 3:**
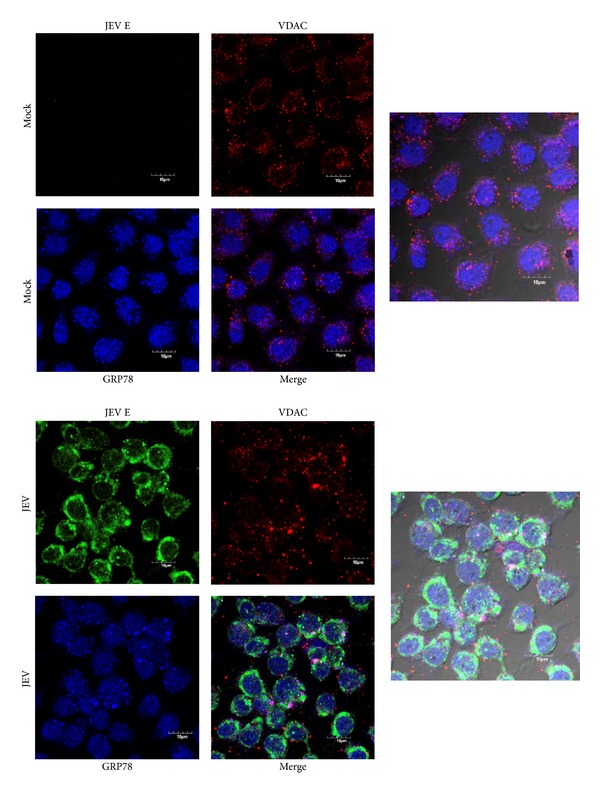
Location of JEV E protein, VDAC, and GRP78 in JEV infected C6/36 cells. C6/36 cells grown on glass cover slips were mock infected or infected with JEV and examined for location of JEV E protein (green), VDAC (red), and GRP78 (blue) using an Olympus FluoView 1000 confocal microscope. Representative individual and merged images are shown, and one panel has bright field added.

**Figure 4:**
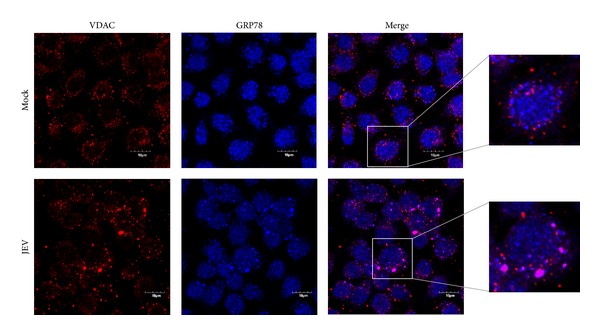
Location of VDAC and GRP78 in JEV infected C6/36 cells. Signal of VDAC and GRP78 extracted from Figure 3 for improved visualization purposes with enlargement.

We next determined whether there was intracellular colocalization between VDAC and GRP78 in both uninfected and JEV infected C6/36 cells. C6/36 cells were either mock infected or infected and at 24 hours after infection they were permeabilized and examined for localization under a confocal microscope. A triple staining technique was employed, with primary antibodies directed against JEV E protein, VDAC, and GRP78. As can be seen in Figure 3, there was significantly increased colocalization between GRP78 and VDAC in infected cells as compared to uninfected cells. For easier visualization, the signals for GRP78 and VDAC only are shown in Figure 4. Analysis of colocalization showed that there was a significant increase in colocalization between GRP78 and VDAC in infected cells (Pearson correlation coefficient 0.63 ± 0.069) as compared to mock infected cells (Pearson correlation coefficient 0.47 ± 0.067; *P* < 0.01).

**Figure 5:**
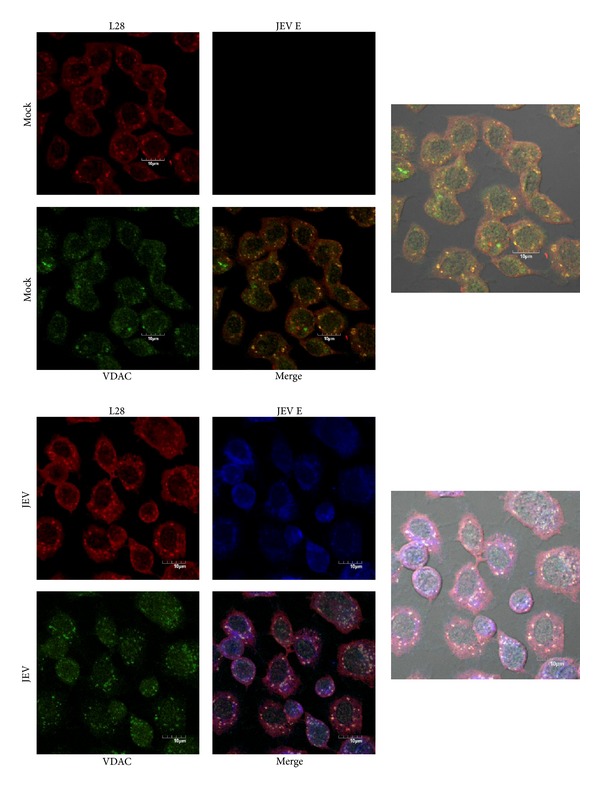
Location of JEV E protein, VDAC, and ribosomal L28 protein in JEV infected C6/36 cells. C6/36 cells grown on glass cover slips were mock infected or infected with JEV and examined for location of JEV E protein (blue), ribosomal L28 protein (red), and VDAC (green) using an Olympus FluoView 1000 confocal microscope. Representative individual and merged images are shown, and one panel has bright field added.

**Figure 6:**
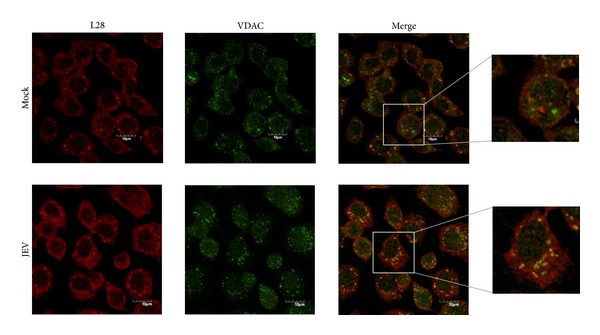
Location of JEV E protein, VDAC, and ribosomal L28 protein in JEV infected C6/36 cells. Signal of VDAC and ribosomal L28 protein extracted from Figure 5 for improved visualization purposes with enlargement.

GRP78 is predominantly localized to the ER, while VDAC is predominantly localized to the outer membrane of the mitochondria. The increased colocalization of GRP78 and VDAC in JEV infected cells would suggest a relocalization of VDAC to the ER in response to infection. To determine if this was the case, C6/36 cells were again either mock infected or infected with JEV and at 24 hours after infection they were examined for the localization of JEV E protein, ribosomal protein L28, and VDAC. As can be seen in Figure 5, a significant increase in colocalization of VDAC and ribosomal protein L28 was observed in JEV infected cells (Pearson correlation coefficient 0.58 ± 0.045) as compared to mock infected cells (Pearson correlation coefficient 0.41 ± 0.091; *P* < 0.01). For easier visualization the signals for VDAC and ribosomal L28 protein only are shown in Figure 6.

## 4. Discussion

GRP78, which is also known as BiP (binding immunoglobulin protein), is a multifunctional protein that has been implicated in a wide range of cellular processes [45]. GRP78 is a member of the heat shock protein 70 (HSP70) family of chaperones and has been best characterized as a central mediator of the endoplasmic reticulum unfolded protein response [27–29]. In unstressed conditions, GRP78 binds to and sequesters three UPR regulatory proteins, PERK, ATF6, and IreI, and upon the induction of ER stress by a number of mechanisms including viral infection, PERK, ATF6, and IreI are released from GRP78 leading to the transcriptional regulation of a number of stress response genes [27–29]. In addition to functions in the ER, GRP78 has been identified as a cell surface expressed protein in a number of cell types [24, 30–33], where it acts as a receptor for a number of ligands [43]. GRP78 has been found in association with a number of proteins on the cell surface, including VDAC, the major histocompatibility complex class 1 (MHC-1) and the teratocarcinoma-derived growth factor 1 (Cripto 1) [43].

It was shown that dengue enters into HepG2 cells via GRP78 [24], and independent confirmation showed that downregulation of GRP78 significantly reduced virus entry [34]. Interactions between GRP78 and dengue E protein have been proposed to occur at several steps of the cell cycle, and both cell surface and intracellular interactions between GRP78 and dengue E protein have been documented [24–26].

This study showed that like DENV E protein, JEV E protein is also able to interact with GRP78; however, while GRP78 has been identified as a possible receptor protein for DENV, we found no evidence that GRP78 was able to mediate internalization of JEV to insect cells, either alone or together with VDAC. GRP78 was also excluded as a receptor protein for JEV in a recent study investigating possible JEV receptor proteins expressed by microglial cells [19].

VDAC, also known as mitochondrial porin, is located in the outer mitochondrial membrane (OMM) and maintains the permeability of the outer mitochondrial membrane [46]. VDAC serves to mediate interactions between mitochondria and the rest of the cell by regulating the transport of ions, ATP, and other metabolites [47]. In addition to other functions, VDAC is believed to play a major role in the regulation and execution of apoptosis through its interactions with members of the Bcl2 family of proteins [48], which mediate release of apoptotic proteins present in the inner membranal space. VDAC has been shown to be associated with GRP78 at the cell surface [42–44], but as shown here, VDAC neither alone nor in combination with GRP78 appears to mediate the internalization of JEV.

As noted, VDAC is known to be predominantly localized in the OMM and additionally at the cell surface. We have shown that in response to JEV infection VDAC shows a significant increase in colocalization with the ER resident GRP78. The fact that VDAC relocalizes to colocalize with GRP78 in the ER, rather than GRP78 relocalizing to colocalize with VDAC at the mitochondria, was shown both by the physical alteration of VDAC localization in response to JEV infection and the fact that JEV infection increased colocalization between ribosomal L28 protein and VDAC.

Several studies have previously observed the close localization of VDAC with the ER. Some studies have suggested that this arises from mitochondria physically being in close association with the ER, which may be coupled with relocalization of VDAC within the mitochondria to form VDAC enriched microdomains that are physically in close contact with the ER [49], while other studies have suggested that VDAC itself is physically located within the ER [50]. Either way, our study shows that in response to JEV infection, VDAC is redistributed to be in close contact with the ER in insect cells. As noted, VDAC mediates the release of ions and a number of metabolites including ATP [47], and a recent study with DENV NS3 has suggested that ATP levels may be a critical component in balancing the unwinding and annealing activities of the helicase portion of NS3 [51]. As such, the relocalization of VDAC would suggest that this is an important part of JEV replication in insect cells. Further study may elucidate whether such a mechanism similarly occurs in mammalian cells.
